# Optimizing Chlorogenic Acid Extraction From Spent Coffee Grounds: A Comparative Review of Conventional and Non‐Conventional Techniques

**DOI:** 10.1002/fsn3.70315

**Published:** 2025-06-27

**Authors:** Mariam Ramadan, Abdulmannan Fadel, Abir Bahi, Sedra Alajlani, Mahra Saeed Salem Al Neyadi, Eiman Subaie Hamdan Alshamsi, Sana Naser, Hafiz Muhammad Shahbaz, Rita Samuel, Yazan Ranneh

**Affiliations:** ^1^ Department of Nutrition and Health, College of Medicine and Health Sciences United Arab Emirates University Al Ain UAE; ^2^ Department of Nutrition and Dietetics, College of Pharmacy Al Ain University Al Ain UAE

**Keywords:** bioactive compounds, chlorogenic acid, conventional solvent extraction, green technologies, microwave‐assisted extraction, phenolic extraction, spent coffee grounds, ultrasound‐assisted extraction

## Abstract

Spent coffee grounds (SCGs) pose a significant environmental burden for coffee‐producing industries due to the large quantities generated during coffee brewing. However, SCGs also represent an underexplored and sustainable source of bioactive compounds, particularly chlorogenic acid (CGA), known for its antioxidant, anti‐inflammatory, and antimicrobial properties. This systematic review evaluates and compares various conventional and non‐conventional extraction methods for CGA from SCGs, assessing their efficacy in terms of extraction yield, environmental impact, cost‐efficiency, and processing time, factors often overlooked in earlier studies. Ultrasound‐assisted extraction (UAE) emerged as the most effective technique, yielding the highest quantity of CGA (587.7 ± 46.6 mg/g), using ethanol as a solvent with a solid‐to‐liquid ratio of 1:30. UAE was also identified as the most environmentally friendly technique. In contrast, conventional solvent extraction (CSE) provided a moderate CGA yield (39.5 ± 2.1 mg/g) using water as a solvent and was the most cost‐efficient technique. Microwave‐assisted extraction (MAE) offered the fastest extraction time with a CGA yield of 84 ± 2.8 mg/g. By integrating technical performance with sustainability considerations, this study offers new insights into the optimization of SCG valorization. Furthermore, it supports the advancement of circular bioeconomy strategies through the efficient recovery of high‐value bioactive compounds.

## Graphical abstract

Spent coffee grounds are a rich source of chlorogenic acid, a compound with antioxidant, anti‐inflammatory, and antimicrobial properties. This review conducts a comparative analysis between the conventional and non‐conventional extraction techniques based on CGA yield, environmental sustainability, cost, and processing time. Ultrasound‐assisted extraction (UAE) emerged as the most efficient method, providing the highest CGA yield and demonstrating the best environmental performance.

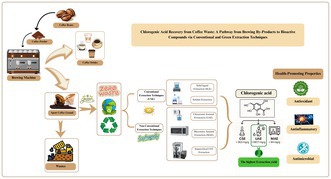

## 1 Overview of Phenolic Extraction From Food Waste

The valorization of food waste through the extraction of bioactive compounds has garnered considerable attention due to its potential health benefits and contributions to environmental sustainability (Sorrenti et al. 2023). Recent studies have highlighted the prevalence of phenolic compounds in various food by‐products, emphasizing their antioxidant properties and potential applications in functional foods and nutraceuticals (Kainat et al. 2022). Furthermore, advancements in extraction technologies, including ultrasound‐assisted extraction, microwave‐assisted extraction, and the utilization of natural deep eutectic solvents, among others, have been explored to enhance the efficiency and sustainability of recovering these compounds from food waste sources (Rifna et al. 2023; Sezer Okur and Okur 2024). These emerging methods aim to optimize yield while minimizing environmental impact, thereby aligning with the principles of green chemistry. Building upon this foundation, our study focuses on the extraction of chlorogenic acid from spent coffee grounds, comparing conventional and green extraction techniques to evaluate their efficacy and sustainability.

## 2 Coffee Production and SCG Waste Generation

**FIGURE 1:**
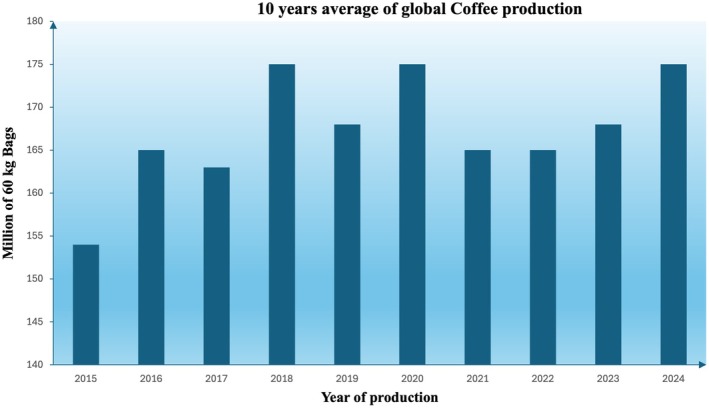
Latest global coffee production trends (USDA 2024).

Coffee is a globally consumed beverage and one of the world's most traded commodities, with an estimated global production of 10.49 million metric tons in 2024 (equivalent ot 174.86 million 60‐kg bags) (Figure 1) (USDA 2024).

Given that global coffee consumption is estimated at around 10.38 million tons per year, according to the USDA and the International Coffee Organization (Beaudor et al. 2023), SCGs represent a significant by‐product of the coffee industry (Zhao et al. 2024). From this, around 11.14 million tons of SCGs are produced annually, which includes 61% moisture content, converting to approximately 6.92 million tonnes of dry material (Beaudor et al. 2023; International Coffee Organization 2023). Disposing of SCGs in landfills leads to environmental concerns, including releasing methane and other greenhouse gases, which significantly contribute to global warming (Iriondo‐DeHond et al. 2019).

Although SCGs are typically treated as waste, they hold significant potential to be repurposed into valuable products. By transforming this biomass, the coffee sector can substantially reduce its environmental impact and contribute to a more sustainable and resilient global coffee industry. Therefore, exploring innovative valorization strategies is crucial for advancing sustainable waste management and supporting the principles of the circular economy.

Actually, SCGs are rich in various polyphenols and antioxidants, such as chlorogenic acids (CGAs) (caffeoylquinic acids) and caffeic acid derivatives (Andrade et al. 2022). Different studies found that caffeoylquinic acid (CQA) and caffeine are the most abundant bioactive compounds (Andrade et al. 2022; Bouhzam et al. 2023; Castaldo et al. 2021; Ho et al. 2020; Makiso et al. 2024). Overall, the reported concentrations of CGAs and caffeine in those studies showed a wide variation, suggesting that origin, coffee variety, roasting degree, brewing procedures, and other different processing conditions influence the bioactive profile of SCGs (Table 1) (Angeloni et al. 2020; Castaldo et al. 2021).

**TABLE 1:** Common compounds found in roasted colombian and roasted arabica spent coffee grounds (mg/100 g dry weight).

Compound Sample	TCQA	diCQA	Caffeic acid	Caffeine	p‐Coumaric acid, ferulic acid, quinic acid	References
Roasted Arabica SCGs from different regions	175.6–489.4	0.6–7	3.9 (Guatemala samples only)	194.1–391.9	Not detected	Andrade et al. (2022)
Roasted Colombian SCGs	209.15 ± 3.19	15.83 ± 1.6	0.72 ± 0.3	119.39 ± 62.3	0.02 ± 0.0/0.08 ± 0.0/0.21 ± 0.0, respectively	Castaldo et al. (2021)
Roasted Arabica SCGs from different regions	155.24–160.98	10.03–14.79	0.58–0.81	107.82–128.41	0.0157–0.0222/0.0698–0.0878/not detected, respectively	Angeloni et al. (2020)

Abbreviations: diCQA, dicaffeoylquinic acid; TCQA, total caffeoylquinic acid.

Notably, caffeic acid was found in low concentrations and only reported for Guatemala samples by Andrade et al. (2022) (Table 1). Also, some phenolic compounds (caffeic acid, ferulic acid, p‐coumaric acid, sinapic acid, and 4‐hydroxybenzoic acid…) were detected by different researchers, highlighting variability in minor compounds among sources (Andrade et al. 2022; Castaldo et al. 2021; Jin Cho et al. 2022; Pyrzynska 2025; Solomakou et al. 2022).

CGAs, a major family of phenolic compounds, are the main class of bioactive compounds in coffee that remain in spent coffee grounds (Solomakou et al. 2022). CGA, chemically identified as 5‐O‐caffeoylquinic acid, is known for its health benefits, including antioxidant, anti‐inflammatory, antimicrobial, and anticancer properties (Changizi et al. 2021; Ekbatan et al. 2018; Lee et al. 2020; Liang et al. 2019; Su et al. 2019). Its potential applications span pharmaceuticals, functional foods, and cosmetics (Iriondo‐DeHond et al. 2019; Liang and Kitts 2015; Su et al. 2019). However, the extraction of CGA from SCGs requires efficient and sustainable methods due to its complex binding within the coffee matrix. As such, researchers have explored both conventional and non‐conventional extraction techniques to maximize CGA yield, reduce processing times, and minimize environmental impacts.

The aim of this review article is to critically evaluate and compare the effectiveness of various extraction methods for CGA from SCGs, categorizing them into conventional and non‐conventional techniques. The review will focus on extraction yield, environmental sustainability, processing efficiency, and cost‐effectiveness. This will provide a comprehensive overview of the existing literature and can provide insights that can guide future research and industrial applications for the recovery of valuable bioactive compounds from coffee waste.

## 3 Methodology

### 3.1 Literature Search Strategy

The literature search for this comparative narrative review was conducted in December 2024 across three electronic databases: ScienceDirect, PubMed, and Google Scholar. We created targeted Boolean search queries and applied them through suitable search fields in each database to retrieve studies about chlorogenic acid (CGA) extraction from spent coffee grounds (SCGs). ScienceDirect searches utilized the default TITLE‐ABS‐KEY field whereas PubMed searches were limited to the title and abstract fields with the [Title/Abstract] tag and Google Scholar required a manual examination of the first 150 results due to its ranking‐based output and lack of field filtering options. The final queries employed were as follows: The research queries included “Spent coffee grounds” AND “chlorogenic acid,” “Spent coffee grounds” AND “extraction” AND “chlorogenic acid,” and “Spent coffee grounds” AND “extraction” AND (“phenolic compounds” OR “polyphenols”). The research queries specifically targeted the core compound of interest (CGA) while also investigating methods for extracting it.

ScienceDirect produced initial search results of about 193, 62, and 118 articles, while PubMed returned 162, 58, and 177 articles for each corresponding query. Three queries produced 450 records for analysis on Google Scholar. Following the removal of duplicate entries and the exclusion of articles without original data or clear methodological explanation, as well as those missing quantitative CGA yield information, researchers identified around 300 unique articles. Two independent reviewers evaluated titles and abstracts for eligibility before examining the full texts of 48 articles. A total of 27 studies met the inclusion criteria for synthesis due to their relevance and scientific rigor, as well as their contribution to evaluating CGA extraction methods from SCGs.

### 3.2 Data Extraction and Standardization

Data were extracted from each article, including CGA yields (standardized to mg/g), extraction methods, solvent types, solid‐to‐liquid ratios, temperature, extraction time, and energy input (for non‐conventional methods). Studies that did not report specific extraction yields or incomplete experimental details were excluded. A total of 27 studies met the inclusion criteria and were presented in Tables 2 and 3.

**TABLE 2:** Conventional extraction methods of CGA from SGCs.

Extraction method	Origin of the spent coffee ground	CGA/CGA derivatives	Solvent	S/L Ratio	Parameters	Outcomes	References
Conventional solvent extraction (CSE)	Robusta and Arabica Varieties coffee spent recovered from espresso coffee machines (Euskovazza S.L. (Usurbil, Gipuzkoa, Spain))	3‐CQA	Ethanol (60%)	1:8	60°C for 2 h with magnetic agitation	0.08933 ± 0.00366 mg/g	García‐Roldán et al. (2023)
			Water		100°C for 1 h	0.143 ± 0.00752 mg/g	
Conventional solvent extraction (CSE)	Spent Coffee Grounds (SCG) obtained from drip bag coffee (Thailand)	CGA	Ethanol (45%)	1:30	75°C, shaking speed 1400 rpm for 20 min	0.369 mg/g	Tangpromphan et al. (2023)
			Water			0.292 mg/g	
Solid–liquid extraction (SLE)	The spent coffee was provided by Misr Coffee (Industrial City, Cairo, Egypt)	CGA	Isopropanol (80%)	—	40°C for 4 h	0.00738 ± 0.00031 mg/g	Badr et al. (2022)
Solid/liquid extraction (SLE)	The spent coffee ground was obtained from the Agriculture Faculty canteen of University of Milan (UNIMI, Italy), composed of different coffee varieties	CGA	Ethanol (70%)	1:100	5–1440 min with continuous stirring	0.592 ± 0.014 mg/g	Abbasi‐Parizad et al. (2021)
Conventional solvent extraction (CSE)	*Coffee arabica* (local branch of a national coffee chain, Ankara, Turkey)	CGA	Methanol (10%)	1:10	50°C for 30 min	0.024 ± 0.0003 mg/g	Okur et al. (2021)
Conventional solvent extraction (CSE)	100% whole bean Arabica coffee (Romania)	CGA	Ethanol/Water/Acetic Acid (50/49.5/0.5, v/v)	1:100	24 h at room temperature, 100 rpm stirring	0.04775 ± 0.00038 to 0.13507 ± 0.00204 mg/g	Vamanu et al. (2020)
Solid/liquid extraction (SLE)	*Coffea arabica* and *C. canephora* var. robusta and roasted coffee blends (local industrial coffee roaster, Italy)	CGA	Anhydrous Ethanol	7:30	Magnetically stirred in the dark for 12 h at 25°C	0.0808 ± 0.0003 to 1.2624 ± 0.0157 mg/g	Balzano et al. (2020)
Conventional solvent extraction (CSE)	Portuguese natural roasted coffee beans Blend of 50% torrefacto roasted coffee and 50% natural Arabica roasted coffee (Spain) Mixture composed by 50% natural roasted coffee and 50% torrefacto roasted coffee	CGA	Ethanol‐water (25:75 v/v)	0.3:25	60°C for 15 min	0.02 ± 0.03 to 22.08 ± 0.03 mg/g	Ramón‐Gonçalves et al. (2019)
Soxhlet extraction (SOX)	Spent coffee from *Coffee arabica*	CGA	Ethanol (60%)	1:25	6 h at constant reflux	0.0252 mg/g	Caballero‐Galván et al. (2018)
Conventional solvent extraction (CSE)	*Coffee arabica* (*AR‐1 and AR‐2*), while the third sample (BL) was a blend comprising various coffee species (Seoul, Korea)	CGA	Methanol (60%)	1:40	60°C for 90 min, shaking at 120 rpm	0.8 ± 0.1 to 3.8 ± 0.1 mg/g	Choi and Koh (2017)
Solid/liquid extraction (SLE)	The coffee was a mixture of Arabica roasted coffees provided by Frellsen Kaffe (Frellsen Rød)	CGA	Ethanol (60%)	1:25	70°C for 10–30 min	30.2 ± 0.1 to 52.3 ± 1.2 mg/g	Burniol‐Figols et al. (2016)
Solid/liquid extraction (SLE)	The SCG were collected from bars using coffee blends richer in Robusta or Arabica, and from used coffee capsules containing regular or decaffeinated coffee (Rome, Italy)	CGA	Ethanol (60%)	1:50	60°C for 30 min with continuous stirring	1.81 ± 0.05 to 6.09 ± 0.11 mg/g	Panusa et al. (2013)
			Water			1.65 ± 0.06 to 6.00 ± 0.11 mg/g	
Conventional solvent extraction (CSE)	Robusta cherry green coffee beans, Mysore, Karnataka, India	CGA	Water	1:4	50°C for 5 min	39.5 ± 2.1 mg/g	Upadhyay et al. (2012)
Conventional solvent extraction (CSE)	Arabica/Robusta blends (Espresso) (Portugal) from different brands	CGA	Water	1:20	Boiled for 5 min with continuous shaking	3.923 ± 1.047 to 5.983 ± 1.462 mg/g	Cruz et al. (2012)
		5‐CQA	Water	1:20	Boiled for 5 min with continuous shaking	0.866 ± 0.224 to 1.75 ± 0.502 mg/g	
Soxhlet extraction (SOX)	Arabica and robusta (Mysore, India)	CGA	Isopropanol: water (60:40 v/v)	1:10	27°C (±2)	0.01145 ± 0.0004 to 0.0161 ± 0.0008 mg/g	Murthy and Madhava Naidu (2012)
Conventional solvent extraction (CSE)	Spent coffee grounds were supplied by NovaDelta‐Comércio e Indústria de Cafés S.A. (Campo Maior, Portugal)	CGA	Methanol (20%–80%)	1:10 to 1:40	30–90 min, agitated at 60°C–65°C	0.37–1.39 mg/g	Mussatto et al. (2011)
Soxhlet extraction (SOX)	Arabica plantation and Robusta cherry (India)	CGA	Methanol	1:8 to 1:12	50°C for 8 h	48.7–56.2 mg/g	Ramalakshmi et al. (2009)

**TABLE 3:** Non‐conventional extraction methods of CGA from SGCs.

Extraction method	Origin of the spent coffee ground	CGA/CGA derivatives	Solvent	S/L Ratio	Parameters	Outcomes		References
Natural deep eutectic solvents (NADES)	Robusta and Arabica Varieties coffee spent recovered from espresso coffee machines (Euskovazza S.L. (Usurbil, Gipuzkoa, Spain))	3‐CQA	Choline chloride: 1,2‐propanediol 50% Choline chloride: 1,2‐propanediol 60%	1:15	65°C for 150 min C in a water bath equipped with a mechanical agitation system	0.12818 ± 0.00218 to 0.13134 ± 0.00075 mg/g		García‐Roldán et al. (2023)
			Betaine: triethylene glycol 60% Betaine:triethylene glycol 70%			0.0717 ± 0.01048 to 0.11609 ± 0.01808 mg/g		
High hydrostatic pressure‐assisted extraction (HHPE)	Coffee Arabica (local branch of a National coffee chain, Ankara, Turkey)	CGA	Methanol	1:10	500 MPa for 15 min at 25°C	0.0812 ± 0.0011 mg/g		Okur et al. (2021)
Ultrasound‐assisted extraction (UAE)				1:10	60% Amplitude for 15 min	0.0850 ± 0.0006 mg/g		
Ultrasound‐assisted extraction (UAE)	*Coffea arabica* L., from Ethiopian origin	(5‐CQA), (3‐CQA), (3,5‐diCQA)	Methanol	1:5	Using ultrasonic bath at a frequency of 40 kHz for 120 min at 20°C	9.78 ± 0.81 mg/g 3.37 ± 0.15 mg/g 0.87 ± 0.06 mg/g		Zengin et al. (2020)
			Water			4.66 ± 0.25 mg/g 2.09 ± 0.15 mg/g 0.85 ± 0.07 mg/g		
			Methanol: Water (50:50)			10.61 ± 0.90 mg/g 2.73 ± 0.20 mg/g 0.75 ± 0.05 mg/g to		
			Ethanol: Water (70:30)			8.62 ± 0.75 mg/g 3.77 ± 0.30 mg/g 1.19 ± 0.09 mg/g		
Microwave‐assisted extraction (MAE)	Spent coffee grounds (Espresso) were collected from the daily waste production of common vending machines, available in the University of Genoa (Italy)	CGA & CGA Derivatives	Ethanol: Water (54:46)	1:10	150°C for 90 min of extraction with a power of 500 W	0.009 mg/g		Pettinato et al. (2019)
			Ethanol: water (32:68)			0.008 mg/g		
Solid–liquid extraction with ultrasound assistance (SLE/UAE)	Espresso spent coffee, provided by local coffee shop (Brazil)	5‐CQA	Water	1:10	stirred by ultrasound equipment for 40 min at an energy of approximately 960 J mL^−1^ at 8°C	3.71 ± 0.40 mg/g		Abrahão et al. (2019)
Supramolecular solvent extraction (SUPRAS)	The coffee variety was Castillo (Circasia, Colombia)	5‐CQA	1‐hexanol 24%, Ethanol 30%, Water 46%	1:5.7	Ambient Tempreture for 1 min	4.3 ± 0.1 mg/g		Torres‐Valenzuela et al. (2019)
Ultrasound‐assisted extraction (UAE)	Spent coffee from Coffee Arabica	CGA	Ethanol (60%)	1:20	50°C for 1 h at 750 W fpower and 20kWh frequency	0.04653 mg/g		Caballero‐Galván et al. (2018)
Pressurized liquid extraction (PLE)	Ten green coffee beans were sourced from Brownhaus Coffee Company (Seoul, Korea), including nine Arabica beans from different countries, and one Robusta bean from Uganda.	5‐CQA	Ethanol (25%–75%)	1:30–1:50	Temperature: 80°C–160°C Time: 5‐20 min, Pressure: 500–2500 psi	51 to 213.98 mg/g		Shang et al. (2017)
Hot pressurized lquid extraction/resin purification process (HPLE‐RP)	—	(3.4‐diCQA), (3‐CQA), (5‐CQA), (3,5‐diCQA), (4,5‐diCQA)	Ethanol (16%)/purification: ethanol (80%)	—	90°C	HPLE: 0.037 mg/g	HPLE‐ RP: 0.00212 mg/g	Mariotti‐Celis et al. (2017)
Ultrasound‐assisted extraction (UAE)	SCG was procured from Chennai, Tamil Nadu, India	CGA	Ethanol	1:05–1:30 g/mL	30°C–50°C for 5–45 min, ultrasonic power output 100–300 W	0.208 to 1.315 mg/g		Al‐Dhabi et al. (2017)
Subcritical water extraction (SWE)	*Coffea arabica* L. grounds purchased from Yunnan Xinbao Coffee Development Co. Ltd. (Baoshan, China).	3‐CQA	Water	1:10–1:70	110°C–190°C for 15–75 min, 5 MPa	0.24 ± 0.03 to 0.98 ± 0.05 mg/g		Xu et al. (2015)
		4‐CQA				0.28 ± 0.00 to 1.36 ± 0.04 mg/g		
		5‐CQA				0.29 ± 0.08 to 1.41 ± 0.01 mg/g		
Microwave‐assisted extraction (MAE)	Robusta cherry green coffee beans, Mysore, Karnataka, India.	CGA	Water	1:4	50°C for 5 min, at 800 W	84 ± 0.28 mg/g		Upadhyay et al. (2012)
		CGA	Methanol			56 ± 1.4 mg/g		
		CGA	Ethanol			49.5 ± 0.7 mg/g		
Ultrasound‐assisted extraction (UAE)	The spent coffee grounds were supplied by “Cantina do CCS”, Brazil	CGA	Ethyl Acetate	1:30	2 h at room temperature, frequency of 55 kHz and potency of 220 V	0.0003 mg/g		Andrade et al. (2012)
Supercritical fluid extraction (SFE)		CGA	CO_2_	—	200 bar/333.15 K	0.0413 mg/g		
		CGA	CO_2_ + 15% ethanol	—	100 bar/333.15 K	0.0196 mg/g		
		CGA	CO_2_	—	300 bar/333.15 K	0.0273 mg/g		

## 4 Chemical Composition of Spent Coffee Grounds (SCGs)

### 4.1 General Composition

SCGs are composed primarily of carbohydrates (up to 50% dry weight), proteins, lipids, and phenolic compounds, including CGA (Lee et al. 2020) (Table 4). Carbohydrates mainly include cellulose and hemicellulose, while proteins constitute about 10%–20% of SCGs (Lee et al. 2020). The lipid content in SCGs accounts for approximately 15%. It is important to highlight that the composition of SCGs is influenced by factors such as coffee species, geographical origin, roasting degree, and brewing method (Matsuda et al. 2022). SCGs also contain some valuable bioactive compounds, such as phenolic compounds, including caffeic acid, ferulic acid, CGA, and other phenolic compounds, making up between 0.3% and 7%, depending on coffee variety and roasting conditions (Su et al. 2019).

**TABLE 4:** The chemical composition of the spent coffee grounds adapted from Battista et al. (2020) and dos Santos et al. (2021).

Chemical compounds of the spent coffe grounds (SCGs) (%)	
Ash	1.3–2.2
Caffeine	0.02
Cellulose	8.6–15.3
Chlorogenic acid	2.3
Fat	2.3
Hemicellulose	31.7–41.7
Lignin	22.2–33.6
Lipids	7–21
Minerals	0.8–3.5
Moisture	74.7
Protein	13–17.54
Tannins	0.02
Total fiber	43
Total pectic substances	0.01

Table 4 displays an approximate content of the chemical compounds that have been identified in the spent coffee grounds (SCGs), noting that the type of coffee beans affects SCG composition, as well as roasting conditions and extraction processes (Battista et al. 2020; dos Santos et al. 2021).

### 4.2 Chlorogenic Acid in SCGs

Chlorogenic acid (CGA), a bioactive phenolic compound with vicinal hydroxyl groups, studied mainly in coffee, which is one of its main sources, but also found in other vegetables such as potatoes, eggplants, apples, and plums (da Costa et al. 2023). The results of the study of Okur et al. (2021) and Andrade et al. (2022) revealed that CGA was the main phenolic compound found in spent coffee grounds. The concentration of CGAs present in spent coffee grounds can be four to seven times higher than their corresponding content in coffee brews (Mitraka et al. 2021). CGA and its derivatives are formed through the esterification of a hydroxycinnamic acid (e.g., caffeic, ferulic, or p‐coumaric acid) and a quinic acid (Frosi et al. 2021; Liang et al. 2019; Oteef 2022).

**FIGURE 2:**
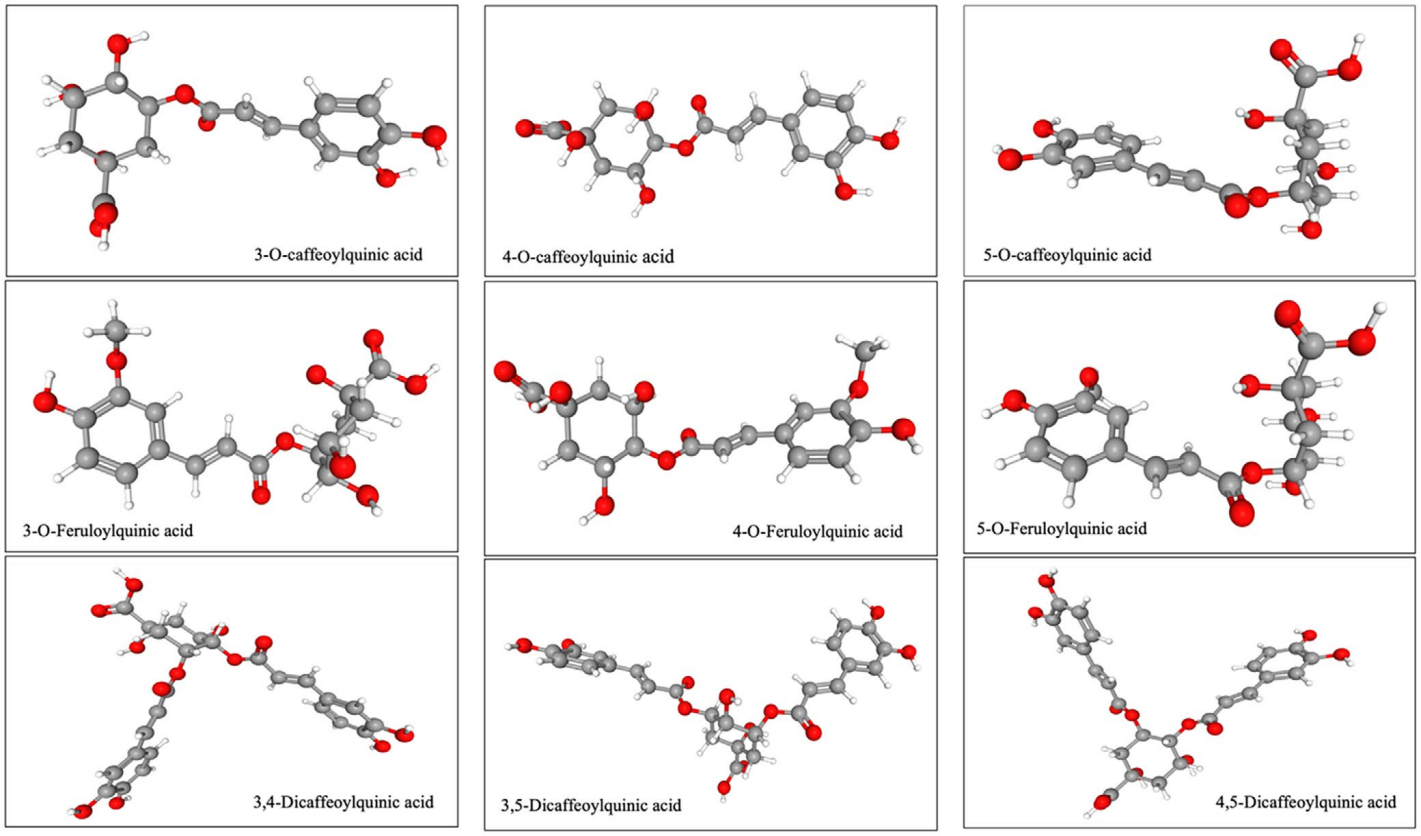
The molecular structures of various chlorogenic acids (CGAs) frequently present in coffee and their common names (Andrade et al. 2022).

The parent structure of chlorogenic acid (caffeoylquinic acid: CQA) is formed by the conjugation of quinic acid (tetrahydroxy‐cyclohexane carboxylic acid) and caffeic acid (3,4‐dihydroxycinnamic acid). Due to the existence of isomers and epimers in the cyclohexane component and variations at the aromatic ring, there exists an entire family of related chlorogenic acids (Liang et al. 2019). Figure 2 illustrates the structures of the typical CGAs commonly found in coffee, along with their corresponding names. In fact, within the CQA subgroup, when one molecule of caffeic acid combines with one molecule of quinic acid, there are three isomers identified as 3‐O‐caffeoylquinic acid (3‐CQA), 4‐O‐caffeoylquinic acid (4‐CQA), and 5‐O‐caffeoylquinic acid (5‐CQA) (Figure 2). However, when two caffeic acid molecules join with one quinic acid molecule, the dicaffeoylquinic acid (diCQA) is formed and three different isomers within the diCQA subgroup exist: 3,4‐dicaffeoylquinic acid (3,4‐diCQA), 3,5‐dicaffeoylquinic acid (3,5‐diCQA), and 4,5‐dicaffeoylquinic acid (4,5‐diCQA), which are common in SCGs and many plant foods (Frosi et al. 2021; Pyrzynska 2025) (Figure 2). When ferulic acid is combined with quinic acid through esterification instead of caffeic acid, the outcome is a feruloylquinic acid. This compound has three isomers, which are identified as 3‐O‐feruloylquinic acid, 4‐O‐feruloylquinic acid, and 5‐O‐Feruloylquinic acid (Figure 2).

Like many phenolics, CGS can bind to proteins, having a high affinity for bovine serum albumin, responsible for loading insoluble hydrophobic compounds in the blood plasma, in addition to scavenging free radicals of oxygen (da Costa et al. 2023; Li et al. 2019; Pan et al. 2019) CGAs are reported to have bioactivities that include antioxidant, anti‐inflammatory, and anti‐colon cancer activities (Ekbatan et al. 2018; García‐Roldán et al. 2023; Li et al. 2019).

## 5 Extraction Techniques for Chlorogenic Acid From SCGs

### 5.1 Overview

Extraction of CGA from SCGs can be classified into two main categories: conventional methods, including solid–liquid extraction (SLE) and Soxhlet extraction (SOX), and non‐conventional methods, such as ultrasound‐assisted extraction (UAE), microwave‐assisted extraction (MAE), and supercritical fluid extraction (SFE). This review analyzes 27 studies detailing various extraction conditions, CGA yields, and the environmental impact of each of the methods, which can be found in Tables 2 and 3.

### 5.2 Conventional Extraction Methods

#### 5.2.1 Solid–Liquid Extraction (SLE)

SLE is one of the most common techniques used for extracting bioactive compounds (phenolic compounds), including CGA, from different plant materials (Alirezalu et al. 2020). SLE consists of placing a solid sample in direct contact with a solvent, enabling the release of phytochemicals into the solvent, which can then be isolated through pressing or filtering the solution (Sharma and Kaushik 2021). A large variety of solvents can be used, such as ethanol, methanol, acetone, or the aqueous phase of solvent blends. Although this method is simple and cost‐effective, it enables the extraction of thermolabile constituents only (Lama‐Muñoz and del Contreras 2022), which requires additional operations, such as solid‐phase extraction or subsequent column chromatography, to remove unwanted residues (Abubakar and Haque 2020). Moreover, too many factors can impact the extraction effectiveness, including the prolonged extraction time, the number of extractions, temperature, the polarity and type of solvent, and the ratio of solvent to sample (Lama‐Muñoz and del Contreras 2022). Despite solid–liquid extraction being a simple and widely used technique, it presents multiple limitations, including prolonged processing time, low extraction efficiency, and the requirement for significant volumes of hazardous organic solvents (Okur et al. 2021). Therefore, new methods and techniques should be employed to address these issues effectively.

#### 5.2.2 Soxhlet Extraction (SOX)

SOX is a continuous solvent extraction technique developed in 1879, also known as hot continuous extraction (Sharma and Kaushik 2021). It requires a specific apparatus that recirculates an organic solvent (pure or mixed in water), at ambient pressure and boiling temperature, over a solid sample, gradually dissolving the target compound (Rocchetti et al. 2019; Yu et al. 2023). Expanding on that, in this process, finely ground plant material is placed in a porous thimble within the Soxhlet apparatus. The heated solvent evaporates, moves into the thimble chamber, condenses, and dissolves the desired bioactive compounds. Once the chamber fills, the enriched solvent flows back into the boiling flask. This cyclic process enables efficient extraction without the need for constant solvent replenishment (Solomakou et al. 2022). Various elements can influence the SOX extraction method, including the volume of solvent in relation to the sample, the temperature used, and whether the mixture is stirred or not (Lama‐Muñoz and del Contreras 2022). The Soxhlet extraction method has several practical advantages that make it popular in research. It uses less toxic solvents and is faster than traditional methods (Yu et al. 2023). A key benefit is the ability to repeatedly rinse the waste matrix with fresh solvent, improving extraction efficiency (Frosi et al. 2021). It also requires less solvent to extract a larger quantity of compounds (Sharma and Kaushik 2021). This technique is suitable for heat‐stable plant materials, does not require filtration, and can handle high temperatures (Abubakar and Haque 2020). Additionally, it is especially useful for materials that are partially soluble or contain insoluble impurities (Abubakar and Haque 2020).

However, Soxhlet extraction comes with some major drawbacks. One of the main issues is the need for high temperatures, which makes it unsuitable for thermolabile plant materials (Abubakar and Haque 2020; Yu et al. 2023). Another limitation is that the process does not allow continuous agitation or stirring, which can reduce the efficiency of extraction (Abubakar and Haque 2020). Additionally, this method is not environment‐friendly, as it releases harmful gases and exposes users to hazardous chemicals, raising concerns about its environmental impact and safety (Okur et al. 2021). Although Soxhlet extraction yields high concentrations of CGA (up to 41 mg/g in some studies), it is solvent‐intensive and time‐consuming, making it less favorable for large‐scale applications (Rocchetti et al. 2019).

These limitations highlight the necessity to develop and adopt innovative, more efficient non‐conventional extraction techniques that are environmental‐friendly and suitable for heat‐sensitive compounds. These techniques should overcome the drawbacks of traditional methods while ensuring higher efficiency and sustainability.

### 5.3 Non‐Conventional Extraction Methods

To minimize energy consumption and processing costs, and to replace harmful solvents with eco‐friendly alternatives, thereby lowering environmental impact, innovative extraction methods such as supercritical fluids (Vandeponseele et al. 2020), microwave‐assisted extraction (Borja et al. 2014), ultrasound (Beaudor et al. 2023; Žlabur et al. 2021), and high‐pressure techniques (Alirezalu et al. 2020) are increasingly favored. These green technologies, which utilize solvents under elevated temperatures and pressures, are designed to enhance extraction efficiency (Mitraka et al. 2021; da Silva et al. 2022).

#### 5.3.1 Ultrasound‐Assisted Extraction (UAE)

Ultrasound‐assisted extraction (UAE) is a non‐conventional technique that utilizes high‐frequency sound (ultrasound) waves exceeding 20 kHz to induce cavitation within the solvent, leading to the rupture of the plant cell wall and enhancing the surface area for solvent absorption. Ultrasounds are able to enhance extraction yields by means of combined mechanisms (sonoporation, fragmentation, deterioration, erosion, sonocapillarity, etc.) (Žlabur et al. 2021). As a result, secondary metabolites will be liberated, and a mass transfer of target compounds into the solvent occurs (Beaudor et al. 2023).

In this approach, plant material is dried, ground into a fine powder, and properly sieved. The resulting sample is then mixed with a suitable extraction solvent and placed in the ultrasonic extractor (Žlabur et al. 2021). The application of high‐frequency sound energy accelerates the extraction by lowering the heat requirements (Abubakar and Haque 2020). There are several factors that can affect the effectiveness of the UAE extraction, such as temperature, sonication time, frequency of the ultrasonic waves, the nature of the sample, and solvent properties (Alirezalu et al. 2020).

Several studies showed mass recovery of bioactive compounds from SCG using the UAE extraction technique (Al‐Dhabi et al. 2017; Valdés et al. 2020; Zhang et al. 2021). Caballero‐Galván et al. (2018) demonstrated that a chlorogenic acid concentration of 46.53 mg/L was obtained through the UAE technique using 60% (v/v) ethanol as solvent, a v/w ratio of 20:1, ultrasonic power and frequency set at 750 W and 20 kWh, respectively, and a constant temperature (50°C ± 2°C) during 60 min. Meanwhile, conventional solvent extraction and Soxhlet extraction produced lower values of chlorogenic acid, 24.87 and 25.22 mg/L, respectively. Recently, Bouhzam et al. (2023) demonstrated that SCG water extraction assisted by ultrasound at room temperature produced the highest quantity of chlorogenic acid and caffeine, yielding 1.15 mg of chlorogenic acid per gram and 0.972 mg of caffeine per gram, respectively. This approach was found to be more effective when compared to the supramolecular solvent and vortex techniques (Bouhzam et al. 2023).

UAE technique provides various advantages, including shorter extraction time, reduced solvent usage, and higher yield, making it suitable for small sample sizes (Al‐Dhabi et al. 2017; Beaudor et al. 2023; Žlabur et al. 2021). However, this technique has some limitations, as it may be challenging to reproduce consistently, and the high energy input during the extraction process may degrade the sensitive phytochemicals by generating free radicals (Abubakar and Haque 2020).

#### 5.3.2 Microwave‐Assisted Extraction (MAE)

Microwave‐assisted extraction (MAE) is one of the innovative green extraction techniques developed for phenolic compounds extraction from different plants. The MAE technique employs microwave radiation to target an object, which can absorb electromagnetic energy in frequencies between 300 MHz and 300 GHz and wavelengths between 1 cm and 1 m (Alvi et al. 2022). This energy is subsequently converted into heat, which facilitates the movement of solvent into the sample matrix and the breakdown of analytes from the sample into the solvent (Coelho et al. 2021). The use of polar solvent leads to dipole rotation and migration of ions, enhancing solvent penetration and assisting the extraction process (Alirezalu et al. 2020). The remarkable selectivity of this technique enables an increase in extraction yields while requiring less time and solvent, potentially tripling outputs in comparison to conventional extraction methods such as SLE (Abdul Mutalib et al. 2024). The common solvents used to extract phenolic compounds from various plant species using the MAE technique are: ethanol, water, methanol, and acetone, but ethanol is preferred for nutraceutical purposes (Frosi et al. 2021). Multiple factors can impact its effectiveness, including microwave power, dielectric properties, duration of extraction, solubility, and characteristics of the solvent (Alvi et al. 2022).

It has been reported that water‐organic solvent mixtures are more efficient than pure solvents for the recovery of CGAs. Based on previous results, the optimal ethanol percentage for CGAs extraction using MAE could be considered in the range of 50%–70% (Abdul Mutalib et al. 2024; Coelho et al. 2021). In a study published by (Solomakou et al. 2022), the MAE technique demonstrated the highest recovery yield (32.2 mg GAE/g d.b.) compared to UAE (18.52 ± 0.52 mg GAE/g dry SCG) and SLE (22.02 ± 0.98 mg GAE/g dry SCG) in the shortest time (5 min). There are significant variations in polyphenol yields from spent coffee grounds (SCG) extracted by the MAE technique, ranging from 7.694 to 31.216 mg/g dry SCG (Ranic et al. 2014), 57.49 mg GAE/g dry SCG (Budaraju et al. 2017), and up to 398.95 mg GAE/g dry SCG (Pavlović et al. 2013). These differences are likely attributed to variations in coffee production and extraction conditions (Solomakou et al. 2022). Upadhyay et al. (2012) demonstrated that MAE could achieve a CGA yield of 84 ± 2.8 mg/g using water as a solvent and a 1:4 solid‐to‐liquid ratio. Microwave‐assisted extraction offers several significant advantages. It allows faster extraction times due to the rapid dielectric heating, which enhances process speed, selectivity, and high extraction performance (Sharma and Kaushik 2021; da Silva et al. 2022). This technique requires less solvent than conventional methods and provides high extraction yields with good reproducibility (Abubakar and Haque 2020; Frosi et al. 2021).

Among the critical limitations of this technique is that it works well for smaller phenolic compounds like gallic acid, quercetin, isoflavin, and trans‐resveratrol, which can endure heating at 100°C for about 20 min, while larger molecules like tannins and anthocyanins are prone to degradation and are not suitable for this extraction (Alvi et al. 2022; Budaraju et al. 2017). One more drawback associated with this method is the low efficiency for volatile substances and the high costs of equipment (Frosi et al. 2021). Additionally, this technique does not support the use of nonpolar solvents, as utilizing them leads to minimal heat generation from the microwave radiation (Alvi et al. 2022).

#### 5.3.3 Supercritical Fluid Extraction (SFE)

A supercritical fluid is any substance that shows both liquid and gas properties at its critical point (Clifford 2018). It behaves like a gas but also retains liquid‐like qualities, allowing it to solvate the sample (Sharma and Kaushik 2021). CO_2_ is generally used as a supercritical liquid in the SFE method, among several other supercritical fluids, such as nitrous oxide, pentane, trifluoromethane, ammonia, ethane, butane, and water (Al Khawli et al. 2019). Carbon dioxide is non‐flammable, non‐toxic (GRAS), and “green” solvent; thus, it has no significant impact on the environment (Roselló‐Soto et al. 2019; Týskiewicz et al. 2018).

Basically, in SFE method, the supercritical fluid is pumped into a cylinder containing the plant material, then the extract laden liquid is collected in a separation chamber (Sharma and Kaushik 2021). SFE produces solvent‐free extracts, as CO_2_ evaporates during depressurization (Týskiewicz et al. 2018). This technique also ensures high‐quality products rich in phenolics and flavonoids with strong antioxidant activity (Alirezalu et al. 2020).

This selectivity can be adapted depending on the compound to be extracted with the use of a co‐solvent, since CO_2_ is not suitable for the extraction of polar phenolic compounds. Ethanol is the most used, because it meets the green technology requirements (Al Khawli et al. 2019). Moreover, carbon dioxide may be more selective when pressure and/or temperature are optimized (Clifford 2018).

There is no recent study for the extraction of phenolic compounds from spent coffee grounds, nor coffee beans, using the SFE method. However, in a previous study, the extraction of green coffee beans with pure CO_2_ as well as with 5% (w/w) of isopropyl alcohol or 5% (w/w) of ethanol as modifiers demonstrated a higher yield of 17% obtained from the extraction with carbon dioxide and ethanol (De Azevedo et al. 2008). Andrade et al. (2012) reported a CGA yield of 41 mg/g using SFE with CO_2_ and ethanol as co‐solvents.

SFE is particularly effective for heat‐sensitive compounds, as it operates at lower temperatures than traditional extraction methods (Sharma and Kaushik 2021). Furthermore, the most important advantage of the SFE technique is its ability to alter the supercritical solvent properties by changing temperature and pressure or by using co‐solvents (ethanol, methanol…) for selective extraction (Clifford 2018; Týskiewicz et al. 2018). While SFE offers excellent selectivity and purity of the extracted compounds, the high cost of equipment and operational complexity limit its widespread industrial application (Týskiewicz et al. 2018).

#### 5.3.4 Subcritical Water Extraction (SWE)

Subcritical water extraction (SWE) is a technique where water is heated to temperatures exceeding its boiling point (100°C–374°C) and is placed under sufficient pressure, up to 22.1 MPa, to remain in a liquid state (typically above 5 Mpa) (Lorenzo et al. 2020; Zhang et al. 2020) to extract bioactive compounds. This technique alters the polarity of the water, allowing it to function like an organic solvent and effectively extract a wide variety of polar and less‐polar bioactive compounds (phenolics, flavonoids, pigments, peptides, and essential oils) without relying on harmful organic solvents (Yabalak et al. 2024). This method is considered a green technology due to its use of water as the primary solvent, reducing reliance on harmful organic solvents (Gbashi et al. 2017; Zhang et al. 2020). Xu et al. (2015) achieved a CGA yield of 72.5 mg/g from SCGs using SWE at 180°C and a solid‐to‐liquid ratio of 1:10. SWE is particularly attractive for industrial applications due to its scalability and environmental sustainability (Ballesteros et al. 2017), under SWE conditions (*T* = 180°C, *t* = 30 min, L/S = 15 mL/g), reported TPC of 36.88 mg GAE, FRAP of 1.0 mmol Fe(II)/g SCG, and DPPH values of 119.02 μmol TE/g SCG. Similarly, SWE extraction of SCGs gives values of 3.4 mg CGA/g SCG for CGA, 22.45 mg GAE/g SCG for TPC, 0.31 mmol Fe(II)/g SCG for FRAP, and 69.31 μmol TE/g SCG for DPPH, under the optimum conditions of 180°C, 30 min, 30 mL/g SCG (Massaya et al. 2023). Recently, the extraction of phenolic compounds from SCGs using the SWE technique produced extracts with higher TPC values (170–331 mg GAE/g DW), especially SWE 150°C, higher DPPH (217–394 mg TE/g DW), and ABTS^+^ (958–975 mg TE/g DW) scavenging activities (Fernandes et al. 2025).

Compared to traditional extraction techniques, SWE has the advantage of utilizing non‐toxic water as a solvent instead of toxic organic solvents, which provides huge environmental benefits. SWE improves extraction efficiency on both polar and non‐polar compounds and reduces extraction time and energy consumption, which considerably reduces the cost of extraction processes, as well as environmental pollution (Aminzai et al. 2024; Yabalak et al. 2024).

#### 5.3.5 High Hydrostatic Pressure‐Assisted Extraction (HHPE)

HHPE is a non‐thermal extraction technique that applies high hydrostatic pressure, in the range of 100–900 MPa, to break down the cell matrix and release bioactive compounds (Okur et al. 2021). In this technique, the application of high hydrostatic pressure enhances solvent penetration into plant cells, increasing cell permeability and promoting the diffusion of bioactive compounds (Alirezalu et al. 2020). This method preserves heat‐sensitive compounds and reduces the need for solvents. Okur et al. (2021) reported CGA yields of 68 mg/g using HHPE at pressures up to 600 MPa, demonstrating the technique's potential for high‐efficiency extraction with minimal environmental impact. In a recent study, it was reported that HHPE extraction using 80% methanol for 15 min at 500 MPa yielded 81.2 mg/g of CGAs and a TPC of 9.42 mg GAE/g (Pyrzynska 2025). Furthermore, a previous study showed that using HHPE (370 MPa, 50°C, 5.7 min) for oil extraction from pepper seeds resulted in higher antioxidant activity, shorter extraction time, and superior oil quality compared to SLE and UAE (Ma et al. 2019). The expensive equipment is the main disadvantage of this technique.

#### 5.3.6 Supramolecular Solvent Extraction (SUPRAS)

SUPRAS extraction involves the use of nanostructured solvents to selectively extract compounds based on polarity (Torres‐Valenzuela et al. 2019). SUPRAS extraction relies on the self‐assembly of amphiphiles (special molecules) to form nanoscale structures that efficiently capture and dissolve target bioactive molecules from complex matrices. Their ability to selectively extract specific compounds, versatility, and eco‐friendliness make them an emerging tool in natural product extraction and food waste valorization (Kfoury et al. 2025). It demonstrates robust performance for polar and nonpolar compounds (Marcinekova et al. 2024). This method is gaining attention for its environmental benefits, as the solvents used are often biodegradable. Torres‐Valenzuela et al. (2019) reported CGA yields of 55 mg/g using SUPRAS, demonstrating the potential for eco‐friendly, selective extraction.

Although SUPRAS is a promising, efficient, affordable, and eco‐friendly extraction technique with wide potential uses, it still has some limitations, like challenges with volatile compounds and lacking cleanup steps; more work is needed to improve the method (Marcinekova et al. 2024).

#### 5.3.7 Natural Deep Eutectic Solvents (NADES)

NADES‐based extraction is a green method that uses naturally derived solvents such as sugars, polyalcohols, organic acids, and amino acids (García‐Roldán et al. 2023). NADES are created by combining hydrogen bond acceptors (HBAs) with hydrogen bond donors (HBDs) (da Silva et al. 2024). The strong hydrogen combination lowers the melting point of the mixture, resulting in a stable liquid solvent that remains at room temperature. The supramolecular structure gives NADES the ability to partially disrupt plant cell walls by the formation of hydrogen bonds between cell wall constituents and the eutectic mixture (Chen and Mu 2019; Liu et al. 2018; Zdanowicz et al. 2018). This technique is more suitable for thermolabile compound extraction (García‐Roldán et al. 2023).

Recently, NADES have been successfully used for the extraction of bioactive compounds from (Ahmad et al. 2021; Fanali et al. 2020; Oomen et al. 2020; Yoo et al. 2018; Zdanowicz et al. 2018). These studies found that betaine:triethylene glycol‐based NADES was the most effective in polyphenol extraction from SCG (Fanali et al. 2020). García‐Roldán et al. (2023) achieved a CGA yield of 48 mg/g using NADES, highlighting the method's sustainability and potential for industrial‐scale application. The combination of DES citric acid and mannitol showed the highest efficiency in extracting TPC from SCGs under the conditions of 10% water, 80°C, and a solid–liquid ratio of 1:15 (w/w), yielding 1620.71 ± 3.72 mg GAE/L (C. N. da Silva et al. 2024). This combination also demonstrated the greatest antioxidant activity in all three methods assessed: FRAP at 1.071 ± 0.006 mol ascorbic acid/L, DPPH radical scavenging activity at 0.234 ± 0.001 mol TE/L, and ABTS radical cation scavenging activity at 0.319 ± 0.002 mol TE/L (da Silva et al. 2024). NADES extraction of phenolic compounds from coffee husk (50% water, 30 min, and a (1:10) (w/v) ratio) produced a yield of 6.16 mg GAE/g and the concentration of polyphenols of 307.81 mg/L (Maimulyanti et al. 2023).

NADES advantages that make it an interesting alternative to organic solvents include that are inexpensive components, easy to synthesize, biodegradable, recyclable, and eco‐friendly with lower toxicity than organic solvents (da Silva et al. 2024; García‐Roldán et al. 2023). However, combining some HBAs and HBDs can lead to DES with high viscosity, making their use difficult.

## 6 Discussion

The extraction of chlorogenic acid (CGA) from spent coffee grounds (SCGs) presents both a scientific challenge and an environmental opportunity. This review has highlighted several extraction methods, comparing their effectiveness based on key metrics such as extraction yield, time efficiency, cost, scalability, and environmental sustainability. The comparison between conventional and non‐conventional techniques reveals distinct advantages and limitations, emphasizing the need for a nuanced approach to selecting the optimal extraction method depending on the specific goals of the application, be it for industrial‐scale extraction or academic research.

The wide variability in reported CGA contents across different studies can be attributed to several interrelated factors. Variations in raw material characteristics, such as genetic background, bean maturation, agricultural practices, degree of roasting, and storage duration, play a crucial role in determining extraction outcomes (Pyrzynska 2025). Extraction efficiency is further influenced by operational conditions, including solvent type, temperature, and extraction time (Kainat et al. 2022; Rifna et al. 2023). Since CGAs are thermolabile, improper temperature control can lead to significant losses. Bouhzam et al. (2023) demonstrated an 82% decrease in concentration after 8 months of storage, underscoring the critical impact of storage time on compound stability. Moreover, the choice of extraction method significantly affects recovery; green technologies like ultrasound‐assisted extraction (UAE) and microwave‐assisted extraction (MAE) can improve yields but must be carefully optimized to avoid degradation of sensitive phenolics (Pyrzynska 2025). Heightening these challenges is the lack of standardized units and methodologies across studies, which complicates direct comparison. As such, the observed discrepancies emphasize the need for harmonized protocols to ensure reproducibility and preserve the integrity of CGAs during and after extraction.

### 6.1 Yield and Efficiency of Extraction Methods

One of the primary metrics for assessing extraction techniques is the yield of CGA, as it directly reflects the efficiency of the method in liberating bioactive compounds from the complex coffee matrix. Among the methods evaluated, ultrasound‐assisted extraction (UAE) consistently demonstrated the highest yield of CGA (587.7 ± 46.6 mg/g), making it the most effective method for maximizing the recovery of CGA from SCGs. UAE's high yield can be attributed to the cavitation effect, which enhances mass transfer by rupturing the cell walls and facilitating the release of intracellular compounds into the solvent (Caballero‐Galván et al. 2018). The use of ethanol as a solvent in UAE, coupled with an optimized solid‐to‐liquid ratio (1:30), likely contributed to the high recovery rate. This is consistent with findings in other studies that report superior extraction efficiencies when ethanol is used due to its ability to solubilize phenolic compounds effectively (Al‐Dhabi et al. 2017; Beaudor et al. 2023; Ma et al. 2019).

Microwave‐assisted extraction (MAE) also demonstrated a relatively high CGA yield (84 ± 2.8 mg/g) but significantly lower than that achieved by UAE. Despite the lower yield, MAE remains attractive due to its rapid processing time. MAE is well‐suited for applications where speed is prioritized, such as in high‐throughput extraction settings or when processing heat‐sensitive compounds. The ability of MAE to extract high quantities of CGA within a few minutes (often less than 10 min) makes it a competitive alternative, especially in situations where the trade‐off between yield and time efficiency is acceptable. However, the relatively high temperatures associated with MAE pose a risk of thermal degradation of sensitive compounds, which could partially explain the lower yields compared to UAE. This highlights the importance of controlling operational parameters like temperature and microwave power to prevent the degradation of CGA during extraction (Alvi et al. 2022; Coelho et al. 2021).

In contrast, conventional methods such as solid–liquid extraction (SLE) and Soxhlet extraction (SOX) produced moderate yields of CGA (39.5 ± 2.1 mg/g and 41 mg/g, respectively). These methods, while widely used in laboratory settings, suffer from several disadvantages, including long extraction times and high solvent consumption (Rocchetti et al. 2019). The relatively low yields and extended processing times associated with SLE and SOX make them less suitable for industrial‐scale operations, where efficiency and cost are critical factors. However, these methods remain useful for small‐scale extractions, particularly in scenarios where low‐cost solvents like water or ethanol are preferred.

### 6.2 Environmental Impact and Sustainability

The environmental sustainability of an extraction method is increasingly becoming a critical factor in both research and industrial applications. The environmental footprint of extraction processes is determined by several factors, including solvent type, energy consumption, and waste generation. Non‐conventional methods, particularly ultrasound‐assisted extraction (UAE), microwave‐assisted extraction (MAE), and supercritical fluid extraction (SFE), stand out for their relatively low environmental impact compared to conventional methods.

UAE, in particular, is recognized for its minimal solvent use and reduced energy consumption. Since UAE operates effectively at room temperature, it avoids the high energy inputs required for heating, making it one of the most eco‐friendly methods available (Caballero‐Galván et al. 2018). Furthermore, the use of ethanol in UAE, which is considered a green solvent, aligns with the principles of green chemistry. Ethanol is biodegradable, non‐toxic, and renewable, making it a more sustainable alternative to conventional organic solvents such as methanol or acetone, which are more hazardous and difficult to dispose of safely (Lama‐Muñoz and del Contreras 2022; Zhao et al. 2024). The high efficiency of UAE also reduces the need for multiple extraction cycles, further minimizing solvent waste.

Supercritical fluid extraction (SFE) also scores highly on sustainability metrics due to its use of supercritical CO_2_, a non‐toxic, non‐flammable, and renewable solvent (Andrade et al. 2012). The ability of SFE to operate at relatively low temperatures and pressures reduces energy consumption compared to other extraction methods that require significant heating. Additionally, SFE offers the advantage of solvent recovery and reuse, which reduces solvent waste and operating costs. However, the high initial cost of equipment and the need for specialized knowledge to operate SFE systems limit its widespread adoption, particularly in smaller laboratories or facilities.

In comparison, conventional methods like Soxhlet extraction (SOX) have a higher environmental burden due to their long extraction times, high solvent consumption, and significant energy requirements for heating. Soxhlet extraction, while effective in achieving moderate CGA yields, typically involves continuous heating of the solvent, which not only increases energy consumption but also contributes to the emission of volatile organic compounds (VOCs) if non‐green solvents are used. The environmental impact of Soxhlet extraction is compounded by the fact that many conventional solvents are derived from petrochemical sources, making them less sustainable in the long term (Bouhzam et al. 2023; Okur et al. 2021). This is a critical consideration in the context of large‐scale operations where solvent use and disposal costs can significantly affect the overall sustainability of the process.

### 6.3 Cost Considerations

Cost efficiency is another important factor when comparing extraction methods, particularly for industrial applications. Among the methods reviewed, conventional solvent extraction (CSE) using water as a solvent was identified as the most cost‐effective approach, yielding 39.5 ± 2.1 mg/g of CGA. The use of water, an inexpensive and widely available solvent, significantly reduces operational costs compared to methods that require organic solvents or specialized equipment (Zhang et al. 2020). However, the trade‐off is the lower yield of CGA and the extended extraction time, which may not be suitable for high‐throughput industrial processes. While CSE remains a viable option for small‐scale or cost‐sensitive operations, it may not meet the efficiency demands of large‐scale CGA recovery from SCGs.

In contrast, non‐conventional methods such as UAE and MAE, while more expensive in terms of initial equipment costs, offer better long‐term cost efficiency due to their shorter extraction times and higher yields. UAE, for example, requires relatively simple equipment, and its operational costs are primarily associated with energy consumption and solvent use. Given its high yield and low solvent consumption, UAE is likely to be cost effective in the long run, particularly in large‐scale applications where high recovery rates can offset the initial capital investment.

MAE also offers significant cost savings due to its rapid processing times, which reduce labor and energy costs. However, the higher risk of thermal degradation and the need for precise control over operational parameters may increase maintenance costs, particularly in sensitive applications where CGA quality must be preserved (Budaraju et al. 2017; Coelho et al. 2021). In industrial applications where throughput and consistency are key, MAE's speed advantage may justify the higher upfront costs, especially in processes requiring quick turnaround times.

Supercritical fluid extraction (SFE), while offering high selectivity and purity of extracted compounds, is typically associated with higher operational and capital costs due to the need for high‐pressure equipment and specialized knowledge to operate the system (Andrade et al. 2012). However, the ability to reuse CO_2_ as a solvent mitigates some of these costs, and SFE may become more economically viable as the technology becomes more widespread and equipment costs decrease. For industries focused on sustainability and green extraction methods, the long‐term cost savings and environmental benefits of SFE could outweigh the higher initial investment.

### 6.4 Scalability and Industrial Applications

Scalability is a crucial factor when transitioning extraction methods from the laboratory to industrial‐scale production. Conventional methods such as SLE and Soxhlet extraction are well‐established but may face challenges in scalability due to their longer processing times, high solvent usage, and relatively low yields. In large‐scale operations, these methods would require substantial increases in solvent volumes and extended extraction times, making them less practical for high‐throughput processes.

In contrast, non‐conventional methods like UAE and MAE are more easily scalable, particularly in industries that prioritize speed and efficiency. UAE, with its high CGA yield and low energy requirements, can be adapted for industrial applications with relatively simple modifications to equipment, such as scaling up the size of the ultrasonic bath or horn (Caballero‐Galván et al. 2018). Similarly, MAE's short processing time makes it an attractive option for industries where rapid extraction is necessary. However, the high initial equipment costs for MAE and the risk of compound degradation must be carefully managed during scaling.

Supercritical fluid extraction (SFE), while highly selective and efficient, faces significant scalability challenges due to the high cost of pressure‐resistant equipment and the technical expertise required to operate the system. However, industries that prioritize high‐purity extracts, such as pharmaceuticals and nutraceuticals, may find SFE to be a worthwhile investment despite these challenges. The ability to fine‐tune extraction parameters in SFE allows for the production of highly specialized extracts, which can command premium prices in niche markets (Andrade et al. 2012).

## 7 Conclusion

This review of extraction methods for chlorogenic acid (CGA) from spent coffee grounds (SCGs) demonstrates the growing potential for sustainable and efficient recovery of bioactive compounds from agricultural waste. Ultrasound‐assisted extraction (UAE) emerged as the most promising method due to its high CGA yield, environmental sustainability, and scalability. Microwave‐assisted extraction (MAE) offers significant time savings and moderate yields, making it suitable for applications where speed is critical. Conventional solvent extraction (CSE) remains the most cost‐effective method for small‐scale operations but may not be suitable for high‐throughput industrial applications. Overall, the findings emphasize the importance of selecting the appropriate extraction method based on specific operational goals, whether focused on maximizing yield, reducing environmental impact, or optimizing cost and efficiency.

## Author Contributions

**Mariam Ramadan:** writing – original draft (lead). **Abir Bahi:** writing – review and editing (equal). **Sedra Alajlani:** investigation (equal). **Mahra Saeed Salem Al Neyadi:** investigation (equal). **Eiman Subaie Hamdan Alshamsi:** investigation (equal). **Sana Naser:** investigation (equal). **Hafiz Muhammad Shahbaz:** investigation (supporting). **Rita Samuel:** supervision (supporting). **Yazan Ranneh:** supervision (equal).

## Conflicts of Interest

The authors declare no conflicts of interest.

## Data Availability

The authors have nothing to report.
